# Multi-aged social behaviour based on artiodactyl tracks in an early Miocene palustrine wetland (Ebro Basin, Spain)

**DOI:** 10.1038/s41598-020-57438-4

**Published:** 2020-01-24

**Authors:** Ignacio Díaz-Martínez, Oier Suarez-Hernando, Juan Cruz Larrasoaña, Blanca María Martínez-García, Juan Ignacio Baceta, Xabier Murelaga

**Affiliations:** 1 CONICET, IIPG – Instituto de Investigación en Paleobiología y Geología (Universidad Nacional de Río Negro-CONICET), Av. Roca 1242, General Roca, 8332 Río Negro, Argentina; 2 Universidad del País Vasco UPV/EHU, Facultad de Ciencia y Tecnología, Departamento de Estratigrafía y Paleontología, Bilbao, Apartado 644, E-48080 Bizkaia, Spain; 3 Instituto Geológico y Minero de España—Unidad de Zaragoza, C/Manuel Lasala 44-9B, 50006 Zaragoza, Spain; 4 Laboratory of Paleomagnetism, CCiTUB and CSIC—Institut de Ciències de la Terra Jaume Almera. Barcelona, 08028 Barcelona, Spain

**Keywords:** Ecology, Solid Earth sciences

## Abstract

We present a new locality with at least 880 vertebrate tracks found at the top of a limestone bed from the lower Miocene Tudela Formation (Spain). The trampled surface was formed by artiodactyls that crossed a muddy carbonate accumulated under the influence of water level variations in a palustrine environment. The tracks reflect different types of morphological preservation. The well-preserved tracks have tetradactyl digit impressions caused by both manus and pes, and are the type series of a new artiodactyl ichnotaxon, *Fustinianapodus arriazui* ichnogen. nov. and ichnosp. nov. The rest of the tracks, which are not as well preserved, are didactyl and were classified as undetermined artiodactyl tracks. According to their preservation, morphology, size, arrangement and orientation, we propose that this tracksite is the product of a social behaviour, particularly gregariousness, of a multi-age group of artiodactyls ~19 Ma ago. The morphologic and palaeoecologic data presented here suggest that the trackmakers were a group of anthracotheres with a livelihood similar to current hippos. They crossed, periodically, a fresh water palustrine area along some preferential pathways (trails).

## Introduction

The Artiodactyla is a large order of placental mammals which includes pigs, peccaries, hippopotamus, camels, giraffes, deer, cows, antelope and sheep^1,2^. The oldest fossil artiodactyls are from the lower Eocene in North America, Europe and Asia, and evolved to the large land mammals of today^3^. They are “even toed” or “cloven hoofed” ungulates because the autopod axis of symmetry is between the third and fourth digits^2^. They normally have autopods with two or four digits, although several basal members presented pentadactyl mani^4^. Artiodactyls occupy a wide range of habitats, including tropical rain forest, temperate deciduous and evergreen forests, prairie, steppes, deserts and mountainous regions, and are present in all the continents except Antarctica^1,2^. Many artiodactyl taxa live in small social groups or large herds that often have a hierarchy, with the social group size related to body size and feeding behaviour^1^.

The fossilized tracks and trackways of extinct vertebrates offer valuable information about locomotion, behaviour, palaeoecology, substrate conditions, and paleoenvironment^5,6^. Indeed, fossil tracks can also complement information from the osteological record by providing additional data on geographical distributions^7^, first and last occurrences^7,8^ and evolutionary radiations^9^. For instance, the oldest artiodactyl tracks are known from upper Eocene strata of Europe and North America^10–12^. Normally, artiodactyl tracks are didactyl^13,14^, but they may be tetradactyl too^11,15^. They are paraxonic and the impression of digits III and IV appear to be equally important in both didactyl and tetratractyl tracks^16^. Moreover, artiodactyl fossil tracks have been found in all continents except Australia and Antarctica^17^, and in different palaeoenvironments such as aeolian, fluvial, alluvial, palustrine and lacustrine settings^18–25^. Social behaviour has been proposed from some artiodactyl tracksites to take into account the presence of parallel trackways^11,26^.

**Figure 1:**
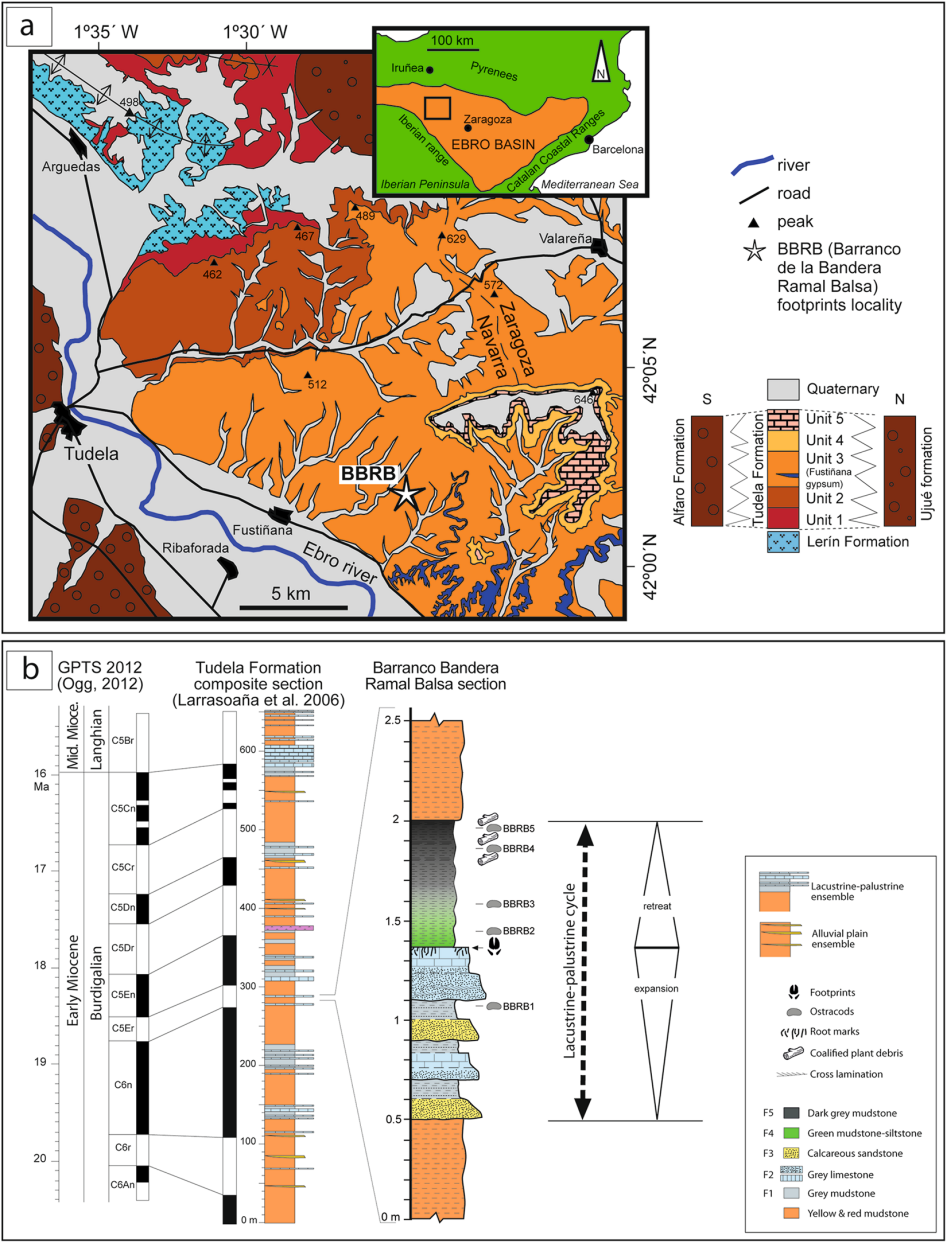
Geographical and geological setting of the Barranco de la Bandera Ramal Balsa tracksite (BBRB) within the Ebro basin. (**a**) Map of the local geology and geography redrawn from Larrasoaña *et al*.^41^. (**b**) Composite lithostratigraphic and magnetostratigraphic logs of the Tudela Formaction stratigraphic section^41^ and their correlation to the GPTS 2012 of Ogg^44^ and to the Barranco de la Bandera Ramal Balsa section. (**a**) and (**b**) originated through Adobe Illustrator CS5, version 15.1.0, www.adobe.com.

Recently, a new tracksite, called Barranco de la Bandera Ramal Balsa (BBRB), has been found with hundreds of artiodactyl tracks in the lower Miocene Tudela Formation (Ebro Basin, Spain) (Fig. 1). The size of the tracksite, the number of tracks and their preservation make it an ideal site to study important aspects of artiodactyls in the past. In this work, an exhaustive description of the track morphology, preservation and ichnotaxomony, as well as a detailed stratigraphic and palaeoenvironmental analysis is provided. Moreover, on the basis of the main orientation, arrangement, shape and size of the studied tracks, a paleoethological reconstruction is proposed.

## Material and Methods

### Vertebrate tracks

**Figure 2:**
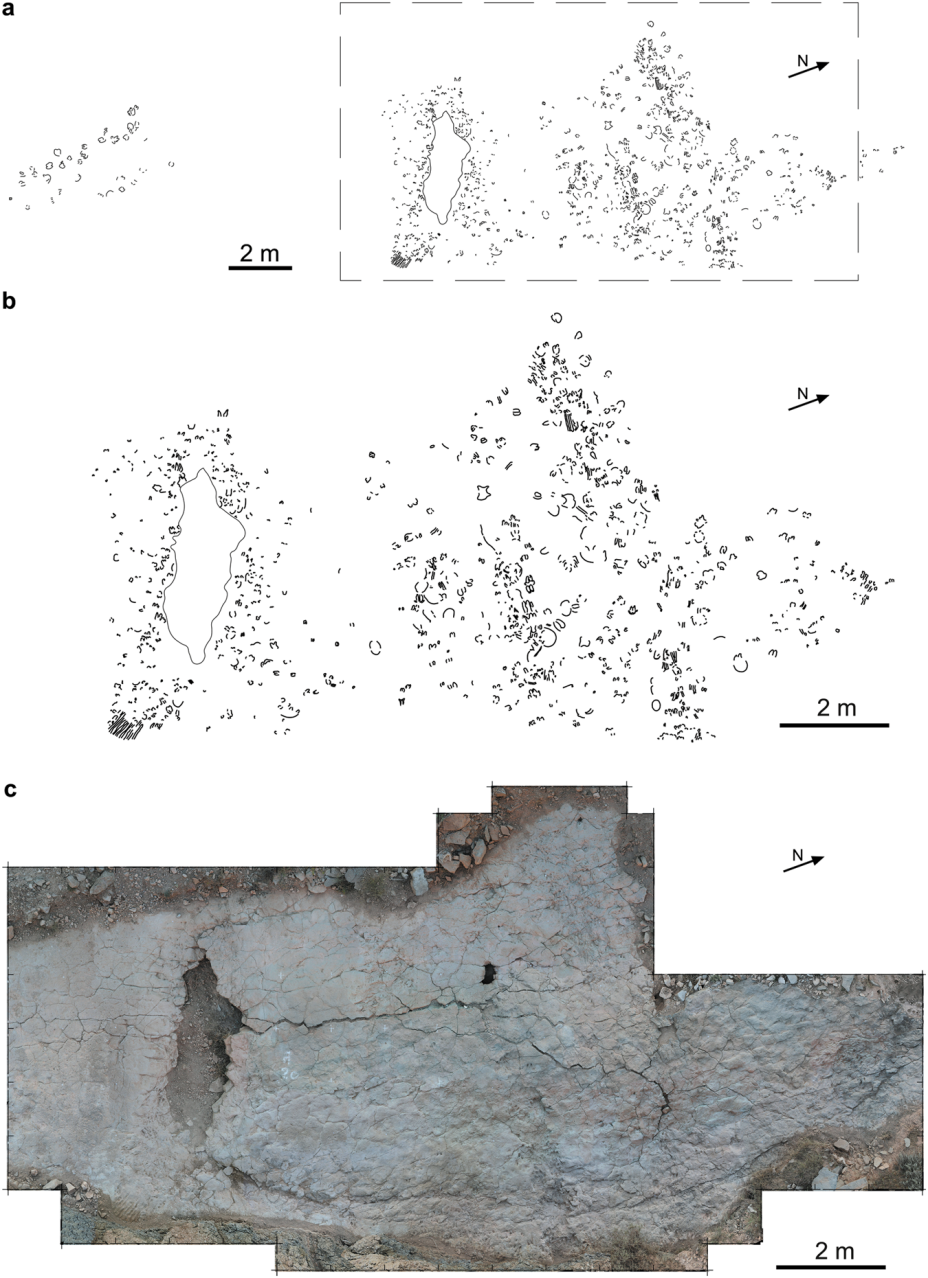
Cartography and orthomosaic of Barranco de la Bandera Ramal Balsa tracksite. (**a**) 2-D cartography of the complete tracksite. (**b**) 2-D cartography of the most trampled section. (**c**) Laser scanner orthomosaic of the most trampled section (FARO Scene 7.1, www.faro.com). (**a**,**b**) originated through Adobe Illustrator CS5, version 15.1.0, www.adobe.com. See Supplementary Information (Supplementary Fig. S1–S3) for quality files.

Field campaigns between 2014–2018 at the BBRB locality produced 2D cartography, photogrammetric models of the most representative tracks, and a laser scanner model which covers most of the tracksite surface (Fig. 2a–c) (see Supplementary Fig. S1–S3). The entire tracksite surface was divided into 1 × 1 m squares and each square was provided with a letter and a number (Supplementary Fig. S2) in order to locate tracks with x- and y-coordinates. Photogrammetric models of tracks 142, 144, 160, 161, 172, 174 and 229 were made with Nikon Coolpix P520 camera with 4.3–7.6 focal length according to the general methodology of Mallison and Wings^27^ and Falkingham *et al*.^28^. Photographs and 3D models are placed in the open repository Figshare as 10.6084/m9.figshare.10072445.v1, 10.6084/m9.figshare.10070303.v1, and 10.6084/m9.figshare.10070252. Point clouds were processed in AgisoftPhotoscan standard version 1.1.4. build 2021 software (http://www.agisoft.ru/). The point clouds were oriented and scaled with the open source Meshlab software (v. 1.3.4BETA https://meshlab.sourceforge.net/). Three-dimensional models were converted to colour maps and contour lines (isolines) in the open source with the software Paraview 4.4.0 version (http://www.paraview.org/).

The main sectors of the site were digitally acquired via the laser scanner FARO Focus3D X 330 HDR. The scans were performed at 360°. In total, 41 stations (18 in the areas with the highest concentration of tracks with a resolution of 3 mm/10 m and another 23 with lower resolution, 7 mm/10 m) were scanned; 15 targets were used for the point clouds registration. The FARO Scene 7.1 software was used to process the data.

**Figure 3:**
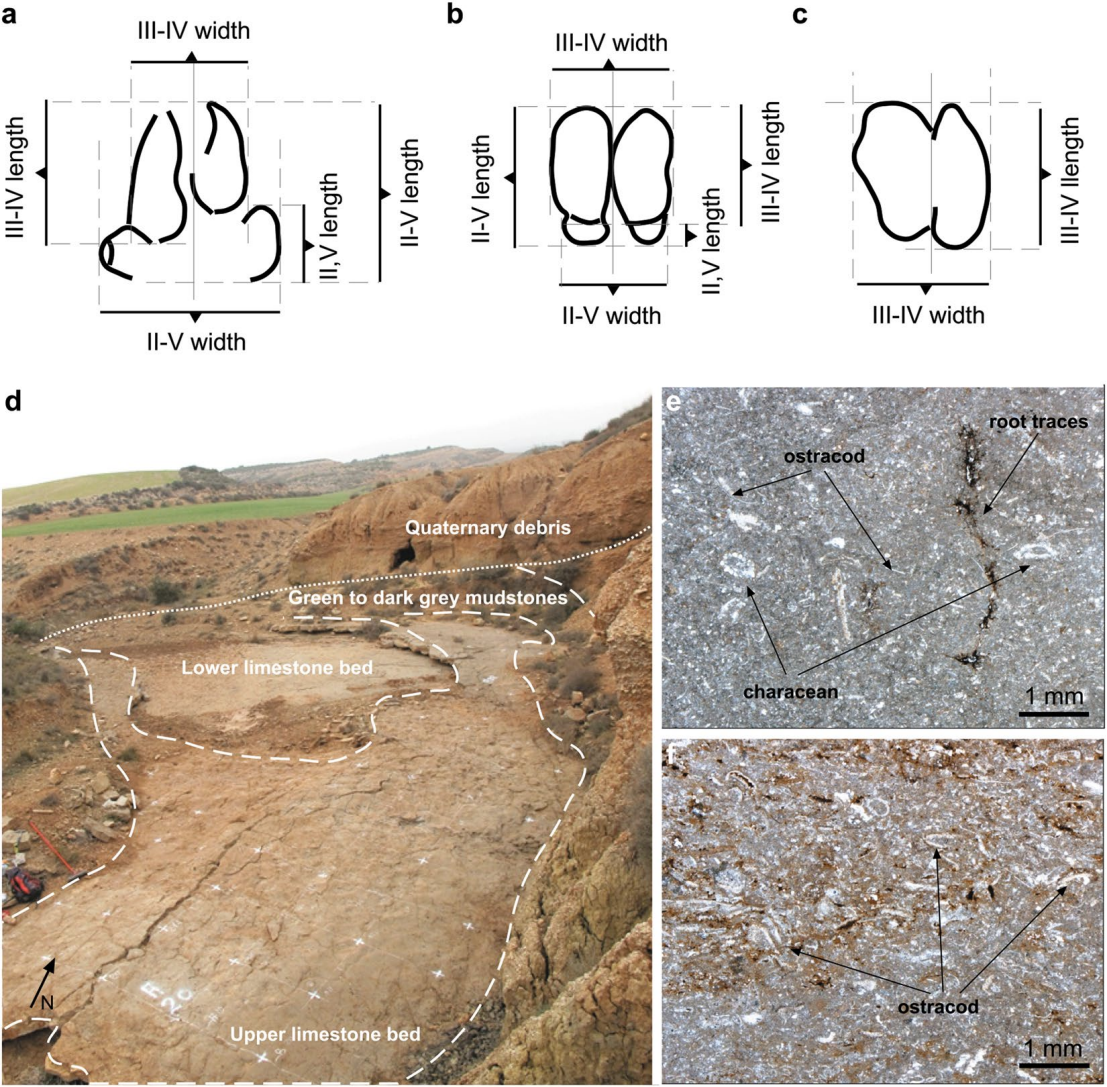
Track measurements and sedimentary facies of the Barranco de la Bandera Ramal Balsa tracksite. (**a**) Digit II-IV and digit III-IV length and width, and digit II, IV length of a tetradactyl manus track. (**b**) Digit II-IV and digit III-IV length and width, and digit II,IV length of a tetradactyl pes track. (**c**) Digit III-IV length and width of a didactyl track. (**d**) Part of the exposed track-bearing surface with the sedimentary facies highlighted by dashed white lines. (**e**) Upper limestone microfacies: Wackestone to fine-grained packstone made of characean and ostracod remains with sub-vertical root traces stained in iron oxides-sulfides. (**f**) Upper limestone microfacies: fine-grained packstone with peloidal matrix rich in ostracod shell debris and disseminations of iron oxides-sulfides. (**a**–**d**) originated through Adobe Illustrator CS5, version 15.1.0, www.adobe.com.

In total, 880 vertebrate tracks have been identified at the BBRB locality. The terminology of Marchetti *et al*.^29^ has been used for the morphological preservation discussions. In the didactyl tracks, the digit III-IV length and digit III-IV width were measured. In tetradactyl tracks, in addition to the above measurements, the digit II-V length, digit II-V width and the digit II,V length (as the length of the lateral digit II and IV) were also estimated (Fig. 3a–c).

The artiodactyl movement patterns were examined by plotting, in a rose diagram, the information of individual tracks due to the fact that there are no clear preserved individual trackways. In tracks where the anterior and posterior surfaces of the track are recognized, the track direction is measured as the angle between the course of the animal’s movement, using the axis of the track that is located between digits III and IV impressions, and the magnetic north. In the tracks where the anterior and posterior surfaces are not clear but the track is almost complete, the track orientation is measured as the angle between the track axis and the magnetic north. In that case, the track shows the orientation of movement, but not its direction. The direction is considered as a Euclidean vector (an arrow) with values between 0° and 360°, while the orientation represents a line with values between 0° and 180°. For the tracks in which it is possible to measure the track direction, the track orientation data are also calculated.

All the aforementioned measurements were obtained from the better-preserved tracks (tracks with a well-defined contour) from the 2D cartography with ImageJ (v. 1.46 R, https://imagej.net/) (Supplementary Table S1).

Statistical analyses (histograms, bivariate plots, rose diagrams, etc.) of these measurements were performed with the open source software Past (v. 2.17c, https://folk.uio.no/ohammer/past/).

### Sedimentology

The paleoenvironmental setting in which the vertebrate tracks were formed was established through sedimentological analysis of the embedding carbonate facies cycle, which forms a 1.5 m thick interval sandwiched between alluvial, fine-grained deposits (Fig. 1b). The study involved the definition of the constituent lithofacies, with their sedimentary structures and compositional-textural attributes, and the analysis of their geometry, vertical stacking pattern and lateral variations. The sedimentological characterization of the indurated lithofacies (limestone and sandstones) also involved the petrographic analysis of 10 representative thin sections. The environmental interpretation was established through integration of the available information and taking previous studies as references on the overall lacustrine-palustrine lithofacies of the Tudela Formation and its laterally-equivalent deposits exposed further east^30,31^.

### Microfossil Extraction

Additional palaeoenviromental information was provided from the analysis of ostracod assemblages extracted from five mudstone intervals above and below the track-bearing limestone level. Samples (500 g. each) were washed and sieved (150 µm mesh size). All adults and last stages of juvenile individuals (A-1 instars) were picked out from the residues. The taxonomic analysis of the ostracod species was based mainly on Meisch^32^ and Karanovic and Lee^3,33^ completed with Fuhrmann^34^ and Mazzini *et al*.^35^. In the five studied samples (BBRB1 to BBRB5) a total of 302 ostracod individuals were identified, representative of ten species and seven genera. Only individuals without evident transport signals, such as valve fractures or abrasions, have been considered.

### Nomenclatural acts

The electronic edition of this article conforms to the requirements of the amended International Code of Zoological Nomenclature, and hence the new names contained herein are available under that Code from the electronic edition of this article. This published work and the nomenclatural acts it contains have been registered in ZooBank, the online registration system for the ICZN. The ZooBank LSIDs (Life Science Identifiers) can be resolved and the associated information viewed through any standard web browser by appending the LSID to the prefix “http://zoobank.org/”. The LSID for this publication is: The LSID for the publication is: urn:lsid:zoobank.org:pub:69962343-FC58-4363-BF90-43085B89D1CF. The electronic edition of this work was published in a journal with an ISSN, and has been archived and is available from the following digital repositories: PubMed Central, LOCKSS.

## Geological Setting

The Ebro Basin, in the northeast of Iberia, was a large continental basin of triangular shape which developed between the Pyrenees and the Iberian and Catalan Coastal ranges during Paleogene and Neogene times^36,37^. It started to form during the late Eocene, coevally to the main phase of the Iberia-Eurasia convergence and the generalized uplift of the Pyrenees, and largely evolved as a closed (endorreic) depression during Oligocene and Miocene times. The sedimentary infill consists of >4,000 m of siliciclastics, carbonates and evaporites arranged in up to eight tecto-sedimentary units (TSU) that record major temporal variations in sediment supply, subsidence and base level^38,39^. During its long depositional history, the general paleogeography of the basin consisted of series of marginal alluvial systems sourced from the emerging mountain reliefs to the north, east and southwest that, through wide alluvial-palustrine plains, converged into central lacustrine areas mainly defined by accumulation of carbonate and evaporitic sediments^38,39^. In the last 20 years, different studies in the northwestern part of the continental Ebro Basin (Fig. 1) have reported a wealth of novel Cenozoic (Eocene to Miocene) palaeoichnological data^40^.

The Tudela Formation, of early to middle Miocene age, comprises the sedimentary succession deposited on the north-western sector of the Ebro Basin during the early to middle Miocene. The main outcrops are located to the east of the town of Tudela, in the area geographically known as the Bardenas Reales of Navarra (Fig. 1a). The stratigraphic unit reaches up to 655 m in composite thickness and is made up of a monotonous succession of yellow to red mudstones and clays with intercalations of discrete channelized sandstones (distal fluvial and alluvial plain deposits), with interbedded intervals of palustrine-lacustrine carbonates and, eventually, evaporites (the Fustiñana gypsum) (Fig. 1b). The Tudela Formation unconformably overlies the Oligocene to lower Miocene Lerin Formation and laterally passes through complex interfingering to the northern and southern marginal coarse alluvial successions of the Ujue and Alfaro Formations^41^. Towards the basin center, to the east and southeast of the reference area, it grades into the carbonate and evaporite-dominated successions of the Alcubierre and Zaragoza Formations^30,38^. The precise age of the Tudela Formation is known from detailed bio- and magnetostratigraphic studies^41–43^, which provided an absolute age range between 20.2 and 15.5 Ma, thus encompassing most of the Burdigalian and the early Langhian stages.

The interbedded lacustrine-palustrine carbonates are the most distinct and best exposed deposits of the Tudela Formation. They occur as intervals of variable thickness and lateral extent made of alternating white to grey limestones and marls, commonly displaying a cyclical vertical facies arrangement indicative of successive stages of expansion and subsequent retreat of lacustrine environments^31^. Up to five main lacustrine-palustrine cycles ranging 20–45 m in thickness and 5–10 km in lateral extent can be recognized within the formation (Fig. 1b). They comprise a wide range of well-stratified lithofacies, from massive marls and nodular limestones with evidence of pedogenesis and episodic wetting and drying (palustrine deposits), to massive and laminated limestones and marls with abundant microfossils (i.e. charophytes, ostracods and gastropods), deposited in open and shallow lacustrine settings, eventually affected by bioturbation and reworking by wind-driven currents and fluvial processes^31^. The Tudela Formation also comprises a myriad of smaller, m-scale facies cycles dominated by carbonate and mixed fine-grained palustrine deposits (Fig. 1b), that likely record ephemeral and localized development of fresh-water ponds and water-logged areas^31^.

The BBRB tracksite analyzed in this study occurs within the small lacustrine-palustrine facies cycle exposed at the Bandera ravine, 6 km to the east of the village of Fustiñana (Fig. 1a). This facies cycle and the embedded vertebrate track level occur within the stratigraphic interval recording the C5Er magnetic reversal^41^, and are thus inferred to have an estimated age of ~18.6 Ma (middle Burdigalian, Ramblian continental stage in Europe)^44^.

## Results and Discussion

### Facies and palaeoenvironmental context

The studied tracks occur within a discrete palustrine facies cycle exposed at the BBRB (Figs. 1b and 3d). It attains a maximum thickness of 1.5 m and occurs sandwiched between packages of yellow to red mottled mudstones, the dominant lithofacies in the Tudela Formation (Fig. 1b). The palustrine facies cycle starts with a marked facies change and comprises four main lithofacies. Additionally, the presence of shells of adult and juvenile ostracods in the same sample supports the assumption that the studied assemblages experienced little transport and can be thus considered as parauthochthonous (good indicator of life environments).

Lithofacies 1. It contains massive to laminated grey mudstones with ostracods and very sparse plant remains and fish teeth (*Palaeoleuciscus* sp.). The ostracod assemblage is dominated by *Ilyocypris bradyi* and *Ilyocypris gibba*, species characteristic of oxygenated and turbulent fresh waters^45,46^. This facies contain discontinuous mm-thick laminae of silt-sized quartz grains and reworked bioclastic debris, and locally shows sparse irregular desiccation cracks and dark haloes by Fe-oxide staining.

Lithofacies 2. It is characterized by massive-nodular to laminated grey limestones, two beds of 14 and 38 cm thick, of dominant wackestone to packstone texture (Fig. 3e,f). The two beds largely consist of micrite with well-preserved remains of ostracod shells and characeans (gyrogonites and poorly calcified stems), plus minor amounts of peloids, silt-sized quartz grains, discontinuous horizontal desiccation cracks and irregular disseminations of Fe oxides and sulfides. The lower half of both limestone levels is sandier in nature, due to the presence of thin discontinuous laminae and concentrations of silt-sized quartz grains, reworked bioclasts and very small intraclasts. In the upper limestone bed, the basal sandy interval also exhibits traces of ripple cross lamination. Both limestone beds display a sharp erosive base and the upper bedding plane also exhibits a very irregular top due to the presence of abundant root marks and subvertical desiccation cracks filled with muddy sediment with disseminated Fe oxides. Laterally, both limestone levels can be traced discontinuously along a distance of about 4–5 km, and finally disappear by gradual facies change to grey-yellowish mudstones. The nodular character and pedogenic features are most conspicuous across these lateral fringes.

Lithofacies 3. A single bed of massive to crudely-laminated, medium to fine-grained, calcareous sandstone. It is grey to brownish in color and laterally varies in thickness between 10 and 15 cm. The sandstone base is sharp and erosive and internally it exhibits crude normal grading and traces of ripple cross lamination. According to composition, it can be classified as a lithic arenite comprising quartz grains, carbonate lithoclasts and minor amounts of reworked bioclasts. The sandstone bed disappears laterally in a few meters, likely as evidence of a lenticular to channel-like morphology.

Lithofacies 4. Massive to poorly laminated greenish mudstones-siltstones in a 30 cm thick interval overlying the upper limestone bed. These facies contain sparse or concentrated plant debris, some small fish teeth (*Palaeoleuciscus* sp.) and ostracod shells, with an assemblage also dominated by *I*. *bradyi* and *I*. *gibba*, both characteristic of oxygenated and turbulent fresh waters^45,46^.

Lithofacies 5. Dark grey, slightly laminated mudstones forming a 35 cm thick interval over the greenish mudstones-siltstones, showing sparse or concentrated plant remains, fish teeth, bone remains and variable proportions of ostracod shells. The ostracod assemblage is dominated by *Pseudocandona albicans*, a genus characteristic of ponded to partially confined waters^41^. The abundance of ostracod shells decreases significantly from base to top.

The described lithofacies are interpreted as representative of an ephemeral shallow lacustrine-palustrine system mainly characterized by the deposition of fine-grained carbonate and mixed muddy deposits, with sedimentary structures indicative of episodic plant colonization, wet and drying processes and incipient pedogenesis. Similar lacustrine-palustrine cycles are common in the Miocene of the Ebro Basin^30,31^, and have been described in other continental Cenozoic successions^47^. The presence of interspersed sandy levels, reworked bioclast laminae and distinct ostracod associations reveal episodic current activity. The interbedded sandstone bed (lithofacies 3) is thought to represent a brief episode of increased fluvial current activity and siliciclastic supply. The vertical succession of facies exposed at BBRB (Fig. 1b) firstly records a gradual change from palustrine to shallow lacustrine conditions (expansion stage), followed by a comparatively faster retreat, likely due to rapid overfilling of the ponded area by increasing siliciclastic supply.

The studied artiodactyl tracksite lies at the irregular top of the upper limestone bed of the lacustrine-palustrine facies cycle (lithofacies 2), on the stratigraphic surface that exhibits the highest concentration of root traces and desiccation features. Visual analysis of the whole outcrop and nearby exposures reveals the absence of similar vertebrate tracks on other bed tops and surfaces, and provides evidence for the relative scarcity and/or poor preservation potential of this association of trace fossils.

### Preservation of artiodactyl tracks

A total of 880 artiodactyl tracks have been identified in the BBRB tracksite. The bedding plane is heavily trampled and hampers identification of individual trackways. The tracks are preserved on the top of an extensive decametric limestone bedding plane and they are covered by laminated mudstones that fill the tracks. Moreover, there is no evidence of layering within the tracks that would suggest the presence of underprints or overtracks^48,49^ in the studied surface. Thus, we consider that all the artiodactyl tracks are probably true tracks^7^ and the surface where they are impressed was the tracking surface^19,49^.

The general shape is variable, being common tetradactyl, didactyl and elongate tracks. They are morphologically affected by substrate consistence and/or taphonomical processes^29,50^. Tracks display four different types of morphological preservation.

**Figure 4:**
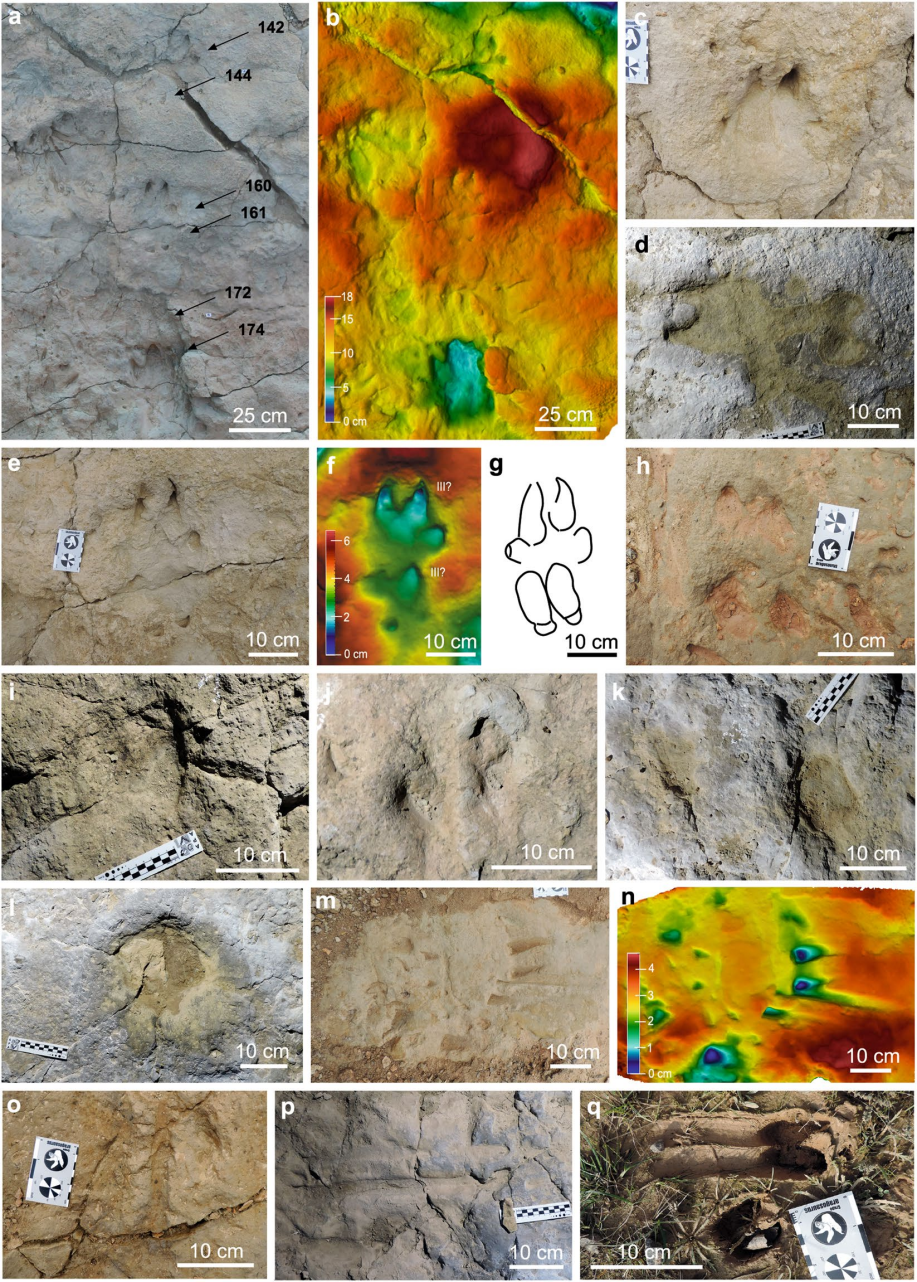
Artiodacyl tracks of the Barranco de la Bandera Ramal Balsa tracksite. Type series of *Fustinianapodus arriazui*: (**a**) Holotypes, tracks 160 and 161 and part of the paratypes, tracks 142, 144, 172 and 174 of the *Fustinianapodus arriazui*; (**b**) False-colour depth map of the (**a**) photogrammetric model (Paraview 4.4.0 version www.paraview.org); (**c**) Paratype, track 412; (**d**) paratypes, tracks 25 (left) and 27 (right); (**e**) Holotypes, tracks 160 (above) and 161 (below); (**f**) False-colour depth map of the holotypes in (**e**) (Paraview 4.4.0 version www.paraview.org); (**g**) Interpretative sketch of the holotypes shown in (**e**) and (**f**). Indeterminate artiodactyl tracks: (**h**) tracks 232 (down) and 233 (up); (**i**) track 379; (**j**) track 76; (**k**) tracks 285 (left) and 290 (right); (**l**) track 236; (**m**) track229; (**n**) false-colour depth map of the (**m**) photogrammetric model (Paraview 4.4.0 version www. paraview.org); (**o**) tracks 409; (**p**) track (297). (**q**) Current slipping (above) and non-slipping (below) sheep tracks.

(1) Well-preserved true tracks: the manus and pes tracks have two well-developed central digit impressions (digit III and IV) and two small lateral ones (digits II and V) in the posterior area. The central digit impressions present a sharp anterior end and rounded posterior surface. The lateral digit impressions are small and laterally projected in the manus tracks and located behind the central digits in the pes tracks. The tracks are clearly impressed and present well-defined contour lines (more pronounced outer margin of tracks) (Fig. 4a–g), and it is possible to identify the track direction. They are equivalent to the grade 2–3 of the numerical scale for quantifying the quality of preservation of vertebrate tracks proposed by Belvedere and Farlow^50^ and Marchetti *et al*.^29^. These authors suggested that the tracks with this grade of preservation are excellent prints upon which to base new ichnotaxa, even at the level of ichnospecies. This type of track is very scarce at the BBRB tracksite (11 tracks, 1.25%, see Supplementary Table S1).

(2) Poorly-preserved true tracks: they are didactyl, preserve only the central digit impressions (digit III and IV), and have an overall well-defined contour line. Some tracks present a sharp anterior end and rounded posterior surface, but in others it is difficult to differentiate between the anterior and posterior surfaces because both are rounded (Fig. 4h–k). Thus, in some tracks it is possible to identify the track direction and in others only the orientation. Moreover, the lack of lateral digit impressions makes it very difficult to distinguish between manus and pes impressions. These tracks are equivalent to grade 1–2 of Belvedere and Farlow^50^ and Marchetti *et al*.^29^, which suggests that they provide good information about the trackmaker and can be related to a previously defined ichnogenus. The poorly preserved true tracks are the most abundant across the tracksite (671 tracks, 76.25%, see Supplementary Table S1).

(3) Very poorly preserved true tracks: they are oval to didactyl shaped tracks with poorly-preserved digit impressions. In some cases it is only possible to calculate the orientation, but not the track direction. The tracks are shallow concavities with a longitudinal axis and poorly-preserved contour lines (not pronounced outer margins of tracks) (Fig. 4l). These tracks are equivalent to the grade 0 of Belvedere and Farlow^50^ and Marchetti *et al*.^29^, which proposed that they indicate only the presence of trackmakers in the area. This type of track is less common at the BBRB tracksite (120 tracks, 13.64%, see Supplementary Table S1).

(4) Slipping true tracks: they are distinctively elongate didactyl tracks, whose width is given by the trackmaker’s autopod width and whose length depends on the degree of slippage. Usually, the slipping tracks have two concave grooves of 2 cm each that are separated medially by an elevation (Fig. 4m–o). In some cases, the grooves present several millimeter-long longitudinal striations that are produced by irregularities in the trackmaker hoof-wall keratin. In addition, some slipping tracks have a non-slipping didactyl track in the end of the slippage grooves and indicates impression of the manus/pes when the trackmaker stopped sliding (Fig. 4p), as in current artiodactyl tracks (Fig. 4q). In this case, it is possible to identify the direction, but in the rest on the slipping tracks only the track orientation is measured. These tracks are equivalent to the grade 1 of Belvedere and Farlow^50^ and Marchetti *et al*.^29^ and are not as common at this tracksite (78 tracks, 8.86%, see Supplementary Table S1).

**Figure 5:**
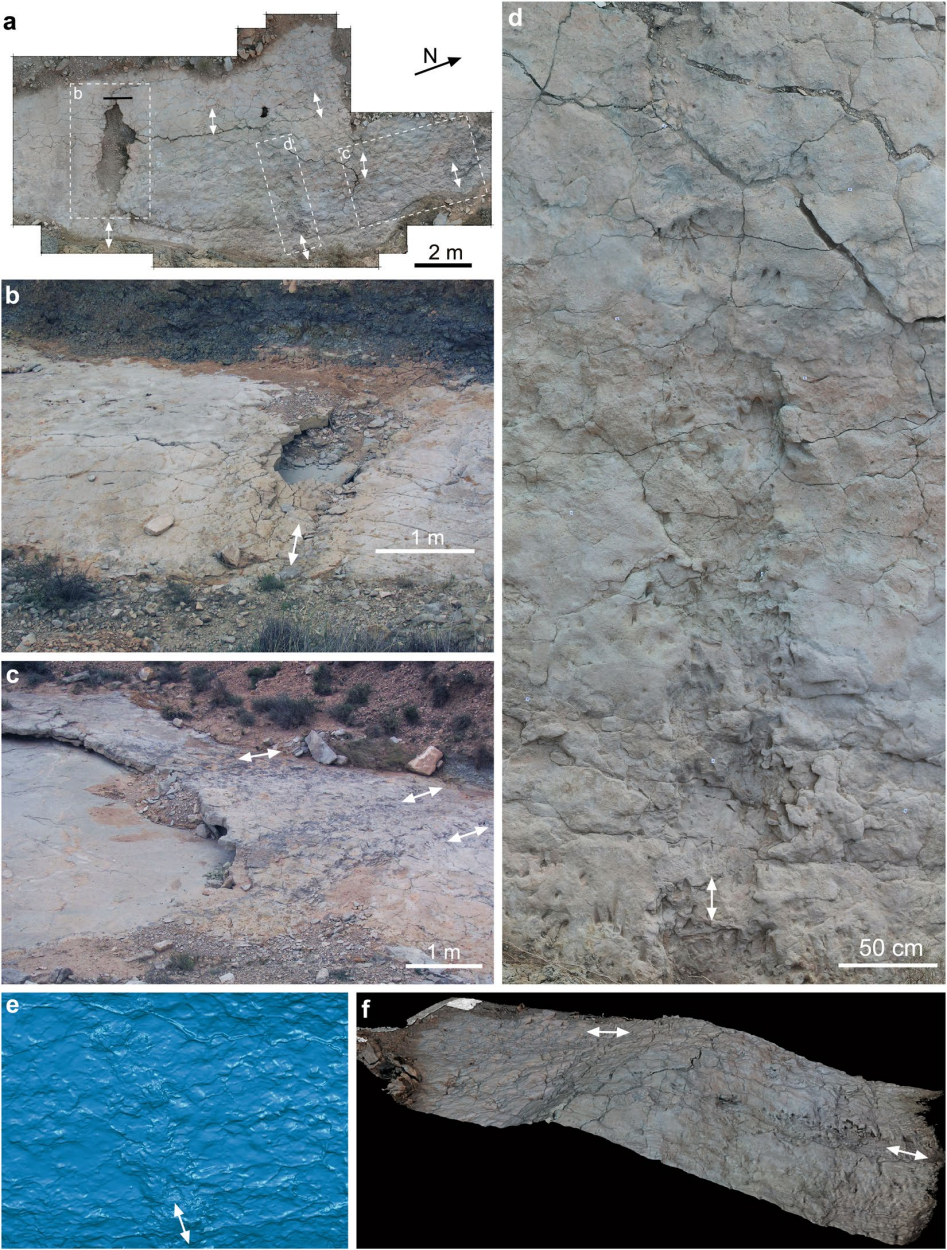
Artiodacyl trails of the Barranco de la Bandera Ramal Balsa tracksite. (**a**) General view of the trampled surface with the main trails indicated by squares with dashed line. (**b**–**d**) Details of artiodactyl trails. (**e**) False-colour view of the laser scanner 3D model of part of (**d**) trail (FARO Scene version 7.1, www.faro.com). (**f**) Textured view of the laser scanner 3D model of the (**d**) trail (FARO Scene 7.1, www.faro.com). White arrows indicate the orientation of the trails.

The tracking surface is separated into areas with heavy, moderate, light or zero trampling degree^51^. The highest concentration (heavy degree) of tracks is located in longitudinal concave depressions (1–1.5 m wide, 0.1–0.3 m depth) that are formed by hundreds of tracks overlapping each other. In the interpretative sketch, only the recognizable tracks were draw although the entire surface was trampled as indicated by the surface roughness. Within these longitudinal depressions, which are considered here as vertebrate trails^52^ (Fig. 5), there are mainly didactyl and slipping tracks. Initially, the substrate must have been moist and pliable, and the continued passage of trackmakers produced a concave depression. Almost all these tracks are didactyl and are part of those considered above as type 2 (taking into account their morphological preservation).The constant trampling compressed the surface and expelled water retained in the substrate. The thickness of the limestone in the central part of this concave depression is much thinner than the thickness of the limestone outside of the trail zone. After trampling, the substrate must have been harder and less moist than before, and subsequent slipping tracks (preservation type 4) were impressed modifying the previous tracks. Off the trail, but relatively close, there are abundant tracks (moderate trampling degree). Some of them are well-preserved, deep and present conspicuous displacement rims (preservation type 1 and 2). Others are poorly-preserved, shallower but present well-preserved contour line (preservation type 2 and 3). Thus, the substrate must have been moist, but got harder and likely dryer when the second round of tracks were impressed. The same parameters befell further away from the trails, where there are fewer tracks but with similar preservation (depth, displacement rims and contour lines). The very poorly preserved true tracks (preservation type 3) are located randomly away of the trails. They are shallow and lack displacement rims, probably impressed when the substrate was dry and stiff to firm. Finally, in the areas where there are no tracks, root traces and desiccation cracks are visible, which suggests either that the substrate was inappropriate for track generation or that the trackmakers avoided that areas (i.e., the vegetation).

It is difficult to propose the temporal history of this tracksite due to the abundance of tracks and preservation types. Nevertheless, some inferences can be made on order of track formation. The slipping tracks are overstepping the tracks that formed the trail. Some very well-preserved tracks crossed over the trail trampling previous tracks. This suggests that, both the slipping and well-preserved tracks have to be later than the formation of the trails. The very shallow true tracks are not trampled or overlapped and could be impressed in a dry substrate before or after the rest of tracks. Therefore, this tracksite includes two overprinted ichnosuites. The first one is characterized by poorly preserved tracks impressed in a moist mud. In the second one, the substrate is dryer than the previous (but still something moist on the surface), due to trampling and/or evaporation, and allowed the preservation of the tracks with greater detail and even slip marks. The track preservation is strongly influence by periodic changes in the water level of the palustrine-lacustrine pond that would provide dry and wet moments.

### Ichnotaxonomy

#### *Fustinianapodus* ichnogenus nov

urn:lsid:zoobank.org:act:8B50D069-62F9-4BF1-9D97-F8A9DB62503A

Type ichnospecies: *Fustinianapodus arriazui*.

urn:lsid:zoobank.org:act:8B50D069-62F9-4BF1-9D97-F8A9DB62503A

**Holotype**: As for the ichnospecies.

**Paratypes**: As for the ichnospecies.

**Type locality**: As for the ichnospecies.

**Type horizon**: As for the ichnospecies.

**Derivatio nominis**: Fustiniana (Fustiñana in Spanish) is the village where the tracks where found; -podus means foot in Greek.

**Diagnosis:** As for the ichnospecies.

#### *Fustinianapodus arriazui* ichnospecies nov

urn:lsid:zoobank.org:act:1817A07E-7E3E-445F-B259-68E0D1096B4E**Holotype:** Tracks 161–160 (manus and pes) of the BBRB tracksite (Fig. 4a,g). They are in situ and there is a precise photogrammetric model of both tracks (10.6084/m9.figshare.10072445.v1).

**Paratypes:** Tracks 27–25 (manus and pes, Fig. 4d), 174–187 (manus and pes, Fig. 4a), 142–144 (manus and pes Fig. 4a), 83–84 (manus and pes) and 412 (Fig. 4c) (10.6084/m9.figshare.10070303.v1).

**Type locality:** Fustiñana (Navarra, Spain). GPS coordinates: 625860.07N; 4656493.48E.

**Type horizon:** Tudela Formation (Ebro Basin) of Burdigalian, Ramblian continental stage in Europe﻿, in age (lower Miocene, MN3)^43^.

***Derivatio nominis*:** The specific epithet of ”arriazui” is dedicated to the retired teacher Manuel Arriazu, who discovered these tracks.

**Diagnosis:** Tetradactyl, paraxonic and subsymmetrical manus tracks that are as wide as or wider than long; central digit impressions are subparallel, longer than wide with acuminate distal end rotated inwardly and rounded posterior surfaces; lateral (crew) digit impression are smaller than central digits, longer than wide, elongated, and projected laterally from the posterior surface of central digit impressions.

Tetradactyl, paraxonic and subsymmetrical pes tracks that are longer than wide; central digit impressions are subparallel, longer than wide with acuminate distal end and rounded posterior surfaces; lateral (crew) digit impressions are smaller than central digits, as long as wide, subtriangular-shaped and located behind the central digits.

**Description:** The tracks assigned to *Fustinianapodus* are the above mentioned well-preserved true tracks. Although all the manus-pes sets are isolated, the tracks 147 m–187 p, 161 m–160 p and 142 m–144 p could be part of a trackway.

The manus tracks are generally in front of or overprinted by the pes tracks (see Fig. 4c). They are longer and thicker than pes tracks (digit II-V length/width in the manus tracks ranges 19.02 cm/20.08 cm; digit II-V length/width in the pes tracks ranges 14.35 cm/11.95 cm; see Supplementary Table S1 for the ichnotype series measurements). The manus tracks are tetradactyl, trapezoidal in shape, with two central digit impressions larger than lateral ones. In the holotype, the digit II-V length and width is 17.85 cm and 18.41 cm, and the average of all the type series is 19.02 cm and 20.08 cm respectively. The digit III-IV impressions are approximately as wide as long (holotype 12.99 length cm and 12.37 cm, mean type series length 12.05 cm width 12.09 cm). The posterior surface is rounded and wider than the anterior one that is acuminate in shape. The medial ridge (area between internal surfaces of central digit impressions) is straight and wide. The lateral digit impressions are smaller than central ones (digit II, V length = 5.81 cm), elongated and longer than wide. They are located in the posterior area of the central digit impressions and projected laterally.

The pes tracks are tetradactyl, rectangular in shape, and have two central digit impressions (digit III-IV) larger than the lateral ones (digit II, V). They are longer than wide (holotype 14.35 cm and 11.95 cm). The digit III-IV are elongated, longer than wide (holotype 11.19 cm and 11.95 cm, average length 14.47 cm width 13.41 cm) and have rounded anterior and posterior surfaces. Medial ridge is very narrow and straight. The lateral digit impressions (digit II, V = 2.44 cm length) are smaller than central ones, and as long as wide. They are subtriangular in shape located with their base close to the posterior surface of each central digit impression.

**Remarks:** The ichnotaxonomy of artiodactyl tracks is very problematic because of the uniformed development of hoofs that are characterized by a wide range of small morphological variations^23,53^. The bibliographic revision of McDonald *et al*.^54^ considered that there exists at least 17 artiodactyl ichnogenera with more than 35 ichnospecies from Eocene through to Pleistocene times. Recent ichnotaxonomic discussions^23,53^ pointed out the difficulties in selecting useful ichnotaxobases that are not conditioned by the locomotion speed, gaits, sediment water-content, etc. In this study, the number of digit impressions in the well-preserved true tracks has been considered as the most important ichnotaxobase, thus *Fustionianapodus* tracks have been compared only with tetradactyl ichnotaxa.

Primarily, *Fustinianapodus* differs from the other tetradactyl artiodactyl ichnotaxa in the shape and position of lateral digit impressions. According to Sarjeant and Langston^12^, *Cervipeda dicroceroires* (Vialov^55^) presents round to rounded-triangular lateral digit impressions. The ichnotaxon *Bothriodontipus* Casanovas and Santafé^15^ has two ichnospecies, *B*. *agramunti* Casanovas and Santafé^15^ and *B*. *rovirai* Santamaria *et al*.^56^. In both ichnospecies, the manus and pes lateral digit impressions are elongated. Nevertheless, *Fustinianapodus* has subtriangular lateral digit impressions in the pes tracks, and elongate digit impressions in the manus tracks. The differences between *Fustinianapodus* and the other Paleogene-Neogene tetradactyl ichnotaxa justify the erection of a new ichnotaxon.

### Indeterminate artiodactyl tracks

**Material:** All other artiodactyl tracks herein except the type series of *Fustinianapodus* (e.g., Fig. 4h–p).

**Locality:** Fustiñana (Navarra, Spain). GPS coordinates: 625860.07 N; 4656493.48E.

**Horizon:** Tudela Formation (Ebro Basin) of Burdigalian, Ramblian continental stage in Europe﻿, in age (lower Miocene)^43^.

**Description:** These tracks refer to the poorly preserved true tracks, very poorly preserved true tracks and slipping tracks (10.6084/m9.figshare.10070252) discussed herein. They are didactyl (digits III and IV) and paraxonic artiodactyl tracks. They are generally longer than wide (average 11.22 cm and 10.68 cm respectively) and range from 4.63–25.18 cm length and from 4.5–26.31 cm width.

The poorly preserved true tracks, such as track 232 and 233 (Fig. 4h), are well impressed, small (<10 cm) to medium (>10 cm <15 cm) in size, with clear contour line and in some of them the anterior (sharply defined) and the posterior (roundly defined) are identified. The very poorly preserved true tracks are oval to didactyl with very shallow contour line, and normally large in size (>15 cm) (Fig. 4l,m). Finally, the slipping track are elongated, with two concave grooves and well impressed. They are variable in length and range between <10 cm to 40 cm (see Fig. 4n–p). For more information, please refer to the “preservation of artiodactyl tracks” section.

**Remarks:** Different artiodactyl taxa leave tracks that differ only in minor details, if at all^12^, and this makes ichnotaxonomic analyses difficult. As previously stated, more than 30 ichnotaxa of artiodactyl tracks have been published and almost all are didactyl (see an updated list in Abbassi *et al*.^53^). Fortunately, the BBRB tracksite has enough tracks to discuss the relationships between preservation and morphology. With the exception of the type series of *Fustiniapodus*, the rest of tracks were modified by trampling or were poorly preserved due to changes in substrate conditions (moist versus dry). The morphological differences among the BBRB tracks are considered here as preservational variations^57^, and were misused by some authors to differentiate among artiodactyl ichnotaxa^14^. Therefore, in this case the shape of these tracks has no ichnotaxonomical sense beyond indeterminate artiodactyl tracks.

### Artiodactyl social behaviour inferred from the studied tracks

In ethology, social behaviour refers, in a broad sense, to any type of interaction between one or more individuals of the same species without evaluating positively or negatively the conduct^58^. Social behaviour is divided into five intraspecific relationships:^59–61^ play, domain of the territory, acquisition of social predominance, sexual behaviour, and gregarious behaviour.

Gregariousness could be defined as the habit of living in groups or herds of individuals of the same species that can be temporary (for refuge, new feeding sites or for sexual aggregations) or permanent^61^. Currie and Eberth^62^ suggested that evidence for gregariousness in extinct taxa comes from five broad sources: (1) skeletal morphology, recognition of adaptations for intraspecific combat, display, and vocalization; (2) bonebed assemblages, presence of monotaxic or monodominant fossil assemblages in a bone bed; (3) tracksites, similar tracks in some parallel trackways with the same direction and speed; (4) phylogenetic inferences, interpretations of gregariousness among extinct animal based on comparisons with their living relatives; and (5) comparison with modern ecosystems, where cooperative behaviour improves success rate in a hunt or protection against an attack.

For identifying social behavior, and particularly gregariousness, within the artiodactyl ichnological record presented here it would be important to first answer some of the following questions: Did all the artiodactyl tracks belong to the same species? Were all of tracks impressed by an artiodactyl social group? Did the animals move together?

**Figure 6:**
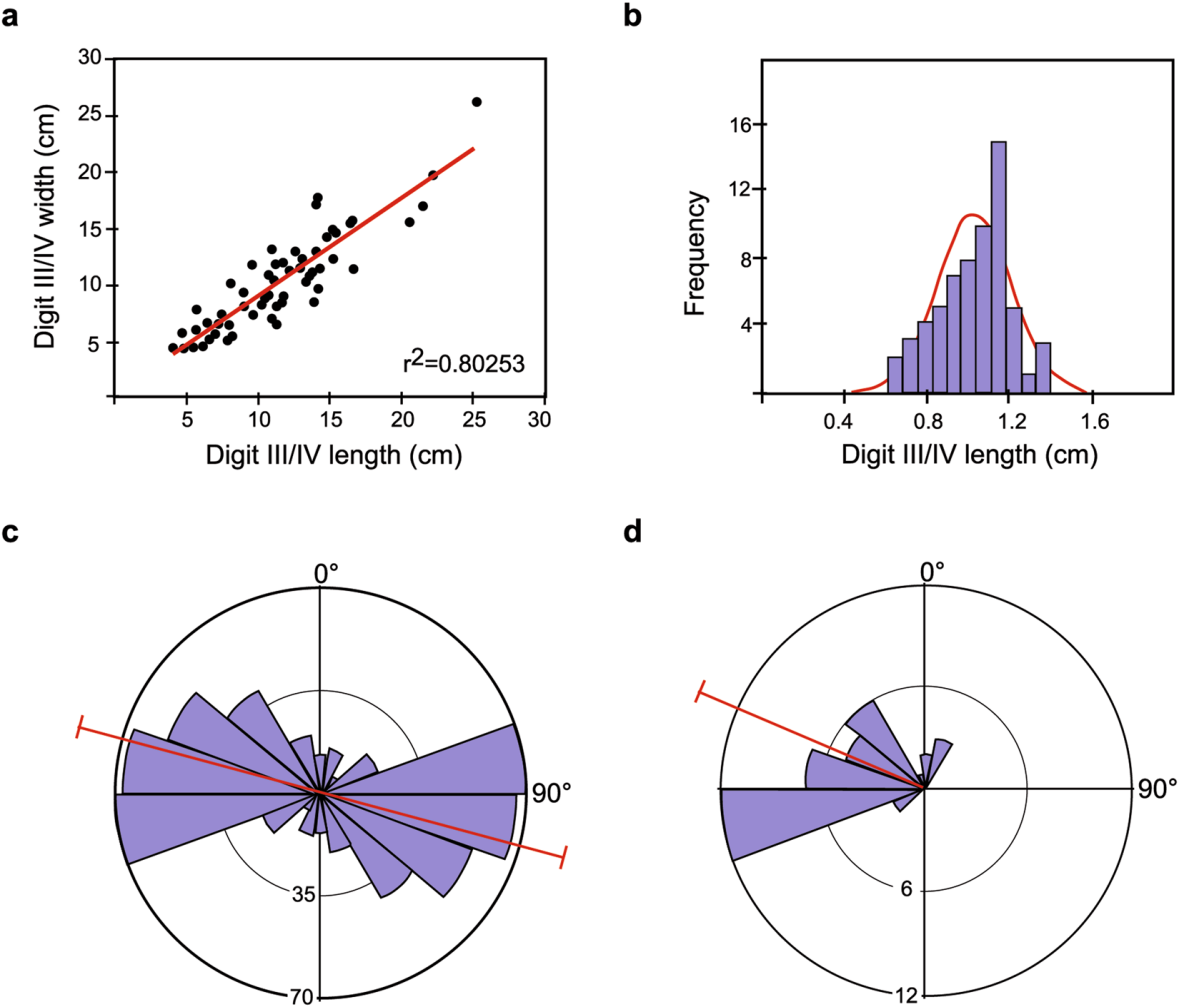
Statistical graphs plotting the artiodactyl track measurements. (**a**) Bivariate graph of track length/track width ratio (n = 63). (**b**) Frequency distribution graph of the normalized data (track length, n = 63). (**c**) Rose diagram with the orientation of the artiodactyl tracks (n = 309). Red line indicates the mean (104.86°). (**d**) Rose diagram with the direction of the artiodactyl tracks (n = 38). Red line indicates the mean (293.34°). All the analyses have been made with the software Past version 2.17c, https://folk.uio.no/ohammer/past/ and drawn through Adobe Illustrator CS5, version 15.1.0, www.adobe.com.

To answer the first question, preservational and morphological information as well as morphometric data must be considered. As previously described, the tracks present different morphologies according to their preservation. These morphological changes (from tetradactyl to didactyl, from clear outline to shallow contour, etc.) do not disprove that the poorly and very poorly preserved tracks, slipping true tracks and the well-preserved tracks are taphonomic variations^57^. Therefore, although the tracks present two different ichnotaxonomic statuses, *Fustinianapodus* and indeterminate artiodactyl tracks, they could be impressed by the same species of artiodactyl crossing a muddy palustrine area influenced by water-level variations, at different moments. Moreover, in order to morphometrically compare these tracks, track length-track width ratio (digit III-IV length/digit III-IV width) was calculated. Most of the studied tracks are not preserved well enough to allow for the measurement of both digit III-IV length and/or width, and therefore a total of 63 tracks using both measurements have been analyzed. The bivariate plot shows that the values have a positive linear relationship (r = 0.89584; r^2^ = 0.80253; p = 0.000; slope = 0.867) (Fig. 6a).

These data show that the length and width are proportional in size and they increase isometrically. A similar arrangement but with different slope values was obtained by Scrivner and Bottjer^18^ for Neogene artiodactyl tracks assigned to three ichnospecies of *Pecoripeda*. They suggested that artiodactyl hoofs stay relatively constant through ontogeny. Thus, different digit III-IV length-width ratio could be related to different trackmakers, and hence, represent different ichnotypes. Based on the morphologic and morphometric data, there is no evidence of more than one kind of trackmaker among the artiodactyl tracks studied herein, and therefore they could be impressed by several individuals of the same taxon.

Alternatively, in order to discuss if the studied sample represent a social group with normal distribution^59^, the normality test has been performed with the track length and width measurements (63 measurable specimens of 880 tracks). Although initially the sample had a skewed distribution, the log10-transformed data illustrate a normal distribution. Normality of the log10-transformed data implies that the sample is unimodal (Fig. 6b), which coincides with the expectation of a single population^63^. Moreover, the track length histogram widely corresponds to a normal curve with most tracks (52 specimens, 82%) between 6 and 18 cm in length. Thus, it is possible that the tracks reflect the known supposed distribution of a multi-aged social group/herd sample with most of the tracks corresponding to medium-sized individuals, and others corresponding to smaller- and larger-sized individuals.

Discussion as to whether the trackmakers moved together, as a group, it is necessary to analyse the orientation patterns of the trackways^64,65^. Since there is no clear evidence of trackways, the angular patterns of individual tracks was studied. The majority of the 309 measured tracks follow an E-W orientation (Fig. 6c). The preservation and the overlapping nature of several tracks on the tracking surface suggest that the tracks were impressed in several moments of passage. Almost all the tracks are arranged in 4–5 delineated areas, considered here as trails, and were used by the trackmakers repeatedly. They compressed the mud, thereby decreasing the bedding thickness due to trampling. On the other hand, the tracks in which it was possible to measure the direction (38 of 880 tracks), show a trend of westward advancement (Fig. 6d). They were probably the last ones to be impressed, just before the surface was covered. The above data suggest that the arrangement of the tracks corresponds to a group of artiodactyls of different sizes (different ages) that passed through the area repeatedly using periodically the same trails.

Of the five sources suggested by Currie and Eberth^62^ to detect gregariousness, the accepted ichnological approach is though the identification of similar tracks in parallel trackways with the same direction and speed. In the BBRB tracksite, although there are no clear trackways, gregarious can be considered because nearly all the tracks impressed by a group of artiodactyls are oriented E-W in specific trample zones and the direction of the best-preserved ones show a westward advance. On the other hand, García-Ortiz and Pérez-Lorente^61^ pointed out that aside from parallel trackways, the accumulation of track and trackways of the same ichnotype, as it is our case, may be considered as evidence of gregariousness. It is possible that this kind of ichnological record has a similar ecological meaning to the presence of monotaxic or monodominant fossil assemblage in a bonebed. Moreover, phylogenetic inferences and ecological factors, as a source of gregarious behaviour, are also evident indirectly with vertebrate tracks. Artiodactyl tracks are presumably impressed by artiodactyl trackmakers and as it is suggested above, many of their extant relatives live in small social groups or large herds^1^, thus it is expected that the extinct artiodactyls present the same social organization.

Overall, we infer that the studied tracksite is the product of a social behaviour, particularly gregariousness, of a herd of one artiodactyl taxon.

### Trackmaker identity

In general, trackmaker identity should reflect the least inclusive group that bounds all taxa sharing similar anatomical characteristics and spatiotemporal distributions^57,66^. The BBRB trackmakers are artiodactyls that impressed didactyl-tetradactyl tracks of 4.63 cm to 25.18 cm in length. As previously suggested, all the tracks were probably made by a group of the same kind of trackmaker, which had at least four functional digits for locomotion. Clifford^4^ studied the anatomy and evolution of manus bones in Artiodactyla and divided them in didactyl, tetradactyl and pentadactyl clades. The pentadactyl ones have a small digit I (not used in the locomotion) and tetradactyl hindlimbs, so that the animals are functionally tetradactyls. They are Cainotheriidae, Anthracotheriidae, Hippopotamidae and Merycoidodontidae. Moreover, the clades Oromerycidae, Tayassuidae, Suidae, Protoceraridae, Leptomerycidae and Tragulidae present tetradactyl fore- and hindlimbs. Among them, the clades Cainotheridae, Anthracotheridae and Suidae with the taxa *Cainotherium*, *Brachyodus*, and cf. *Hyotherium*, respectively, have been identified in several localities from the lower Miocene Tudela Formation^67^. Taking into account the size of the tracks, from 4 cm to 25 cm approximately, it is possible to tentatively discard *Cainotherium* (size of a rabbit) and cf. *Hyotherium* (similar to a wild boar in size) as possible trackmakers^68^. However, the anthracothere *Brachyodus*, similar to a cow in size^69^, is a good candidate to impress tracks of this dimension. Anthacotheres are a fossil group of Cenozoic artiodactyls that ranged in size from that of a small dog to a hippopotamus and which occurred in Eurasia, North America and Africa from the middle Eocene until the late Pliocene^70^. *Brachyodus* as well other medium to large anthracotheres would present hippo-like body proportions, locomotion and lifestyles^71–73^. Ecologically, hippos live in lake and river margin settings, with low topographic gradients^48^, and are usually the only large vertebrate resident in these wetlands^74^. The artiodactyl tracks studied herein are preserved in palustrine limestone beds indicative of extensive wetland areas in the context of a sedimentary basin with negligible topographic differences, and are the unique evidence of vertebrate tracks in this environment. On the other hand, hippos are gregarious and travel in groups with adults that weigh between 1000 kg and 1500 kg in a network of partially submerged trails that are used daily for hippo traffic moving from daytime pools to night time grazing areas^74,75^. These trails, between 0.5 m and 1 m wide and 0.5 m to 1 m deep, lead away from the wetlands and criss-cross adjacent terrain, representing significant topography in this otherwise flat terrain^52,74^. In the BBRB tracksite, a multi-aged group of artiodactyls moved across several well-developed trails of 1 m–1.5 m wide and 0.1 m –0.3 m depth, which represented a significant impact on the substrate (Fig. 5).

**Figure 7:**
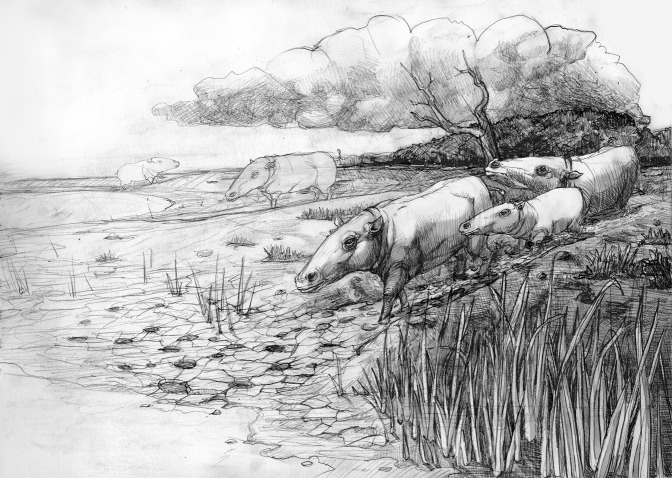
Palaeoecological and palaeoenvironmental reconstruction of the BBRB tracksite. Original drawing by Mauro Pehuén Rosas.

In the ichnological fossil record, the evidence of vertebrate trails is unusual, and is related with the presence of Pliocene-Quaternary hippopotamids^74^. From an evolutionary point of view, Deocampo^52^ pointed out that “*prior to the evolution of the Hippopotamidae, other large semi-aquatic organisms may have occupied a similar ecological niche, and exhibited a similar pattern of interaction with the substrate*”. In this regard, the early Miocene BBRB tracksite could be the oldest record of animals, probably a group of hippo-like anthracotheres, that inhabited this ecological niche and showed a behaviour similar to that of hippopotamids (Fig. 7). The close relationships found here between these artiodactyl tracks and the palaeoenvironment where they were found could be relevant in future ichnofacies studies.

## Conclusions

The artiodactyl tracks studied herein were impressed at the top of a limestone bed that belongs to the Tudela Formation (lower Miocene, Ebro Basin). They present four types of preservation that are mainly conditioned by the characteristics (moisture and compaction) of the muddy carbonate substrate: well-preserved true tracks, poorly-preserved true tracks, very poorly preserved true tracks and slipping true tracks. Ichnotaxonomically, a new artiodactyl ichnotaxon has been defined with the well-preserved true tracks, *Fustinianapodus arriazui*. They are tetradactyl in both manus and pes tracks. The rest of the tracks, which are didactyl, are poorly preserved and have been classified as undetermined artiodactyl tracks. The ichnological record appears to provide complementary evidence to the osteological record and suggests a causative link between locomotion, environment, and behaviour in artiodactyls. The data obtained in this work suggest that the studied tracks were probably impressed by a multi-aged group of monotaxic artiodactyls, which periodically crossed an area using preferred trails. The animals trampled a palustrine area characterized by a muddy substrate and influenced by changes in the water level. Moreover, the tracks are preserved in a palustrine wetland that has a close resemblance to that used by the putative producers, hippo-like anthracotheres.

## Supplementary information

Supplementary Information 1.

Supplementary Information 2.

Supplementary Information 3.

Supplementary Information 4.

Supplementary Information 5.
