# Supplementary figures and images for: Multi-aged social behaviour based on artiodactyl tracks in an early Miocene palustrine wetland (Ebro Basin, Spain)
Source: Sci Rep. 2020 Jan 24;10:1099. doi: 10.1038/s41598-020-57438-4 (PMC6981273; doi:10.1038/s41598-020-57438-4)

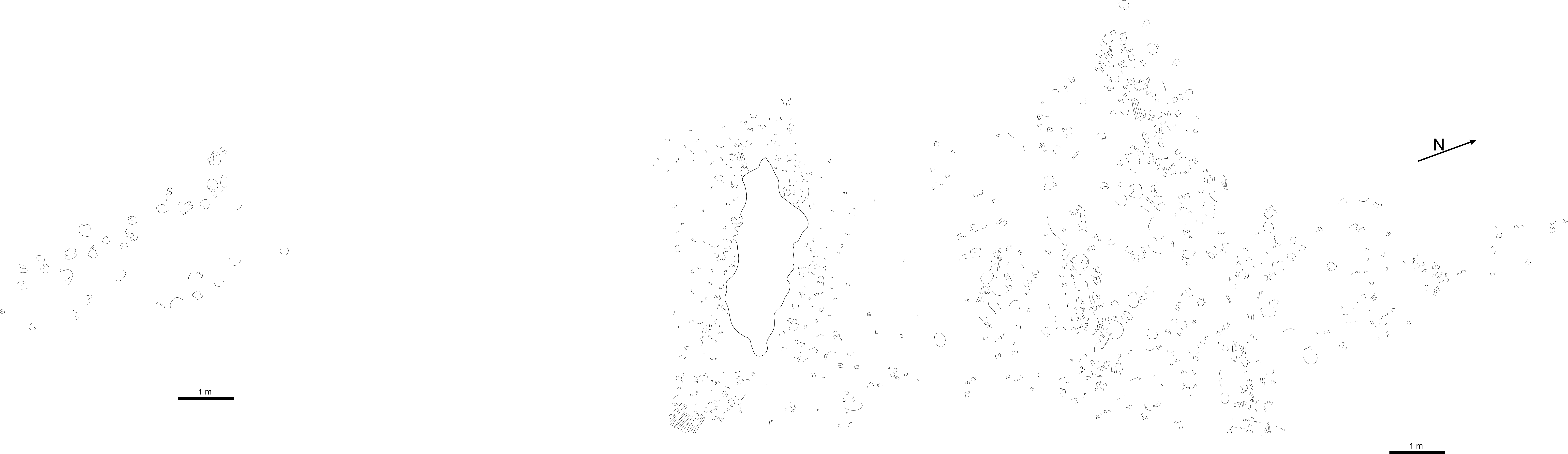

Supplement: Supplementary file 1 — Supplementary Information 1. [file 41598_2020_57438_MOESM1_ESM.tif]

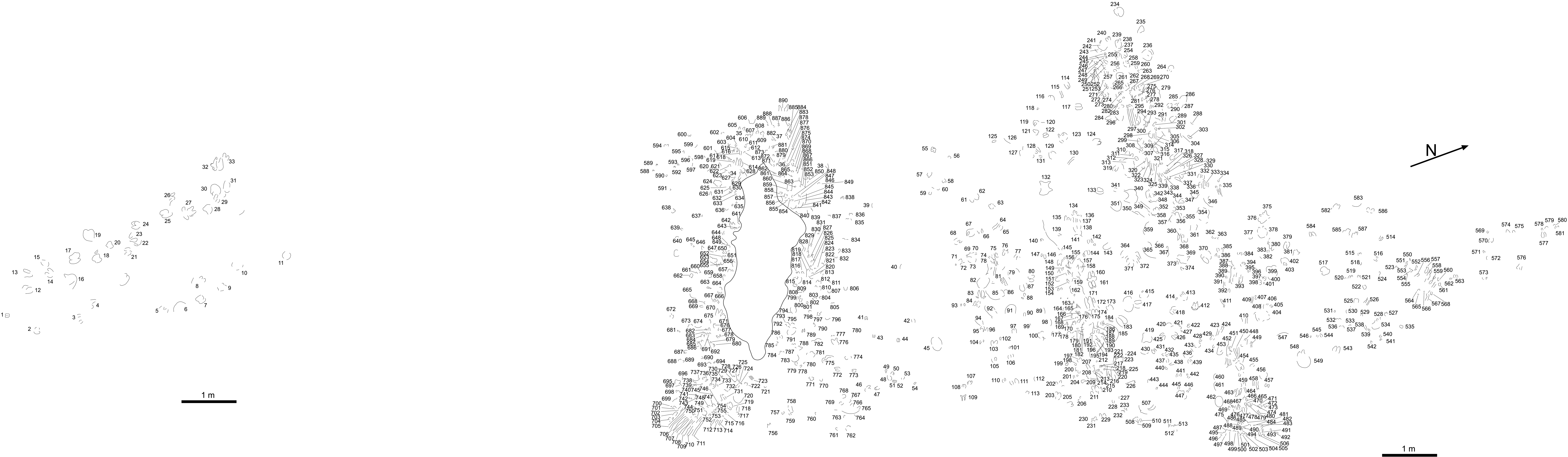

Supplement: Supplementary file 2 — Supplementary Information 2. [file 41598_2020_57438_MOESM2_ESM.tif]
