# Supplementary material for: Multi-aged social behaviour based on artiodactyl tracks in an early Miocene palustrine wetland (Ebro Basin, Spain)
Source: Sci Rep. 2020 Jan 24;10:1099. doi: 10.1038/s41598-020-57438-4 (PMC6981273; doi:10.1038/s41598-020-57438-4)
Supplement: Supplementary file 5 — Supplementary Information 5. [file 41598_2020_57438_MOESM5_ESM.docx]

**Multi-aged social behaviour based on artiodactyl tracks in an early Miocene palustrine wetland (Ebro Basin, Spain).**

Ignacio Díaz-Martínez^a^*, Oier Suarez-Hernando^b^, Juan Cruz Larrasoaña^c,d^, Blanca María Martínez-García^b^, Juan Ignacio Baceta^b^, Xabier Murelaga^b^

^a^ CONICET, IIPG – Instituto de Investigación en Paleobiología y Geología (Universidad Nacional de Río Negro-CONICET), Av. Roca 1242, General Roca. 8332, Río Negro, Argentina

*^b^ Universidad del País Vasco UPV/EHU, Facultad de Ciencia y Tecnología,*

*Departamento de Estratigrafía y Paleontología. Bilbao Apartado 644, E-48080, Bizkaia, Spain.*

*^c^ Instituto Geológico y Minero de España—Unidad de Zaragoza, C/Manuel Lasala 44-9B, 50006 Zaragoza, Spain.*

*^d^ Laboratory of Paleomagnetism, CCiTUB and CSIC—Institut deCiències de la Terra Jaume Almera. Barcelona, 08028, Barcelona, Spain*

* Corresponding author, idiaz@unrn.edu.ar

**Supplementary files**

**Supplementary Fig. S1.** 2-D cartography of the complete tracksite. <https://figshare.com/articles/Barranco_de_la_Bandera_Ramal_Balsa_tracksite/11605059>





**Supplementary Fig. S2.** 2-D cartography of the complete tracksite with the label of each artiodactyl track. <https://figshare.com/articles/Barranco_de_la_Bandera_Ramal_Balsa_tracksite/11605059>

**

**

**Supplementary Fig. S3.** Laser scanner orthomosaic of the most trampled section. <https://figshare.com/articles/Barranco_de_la_Bandera_Ramal_Balsa_tracksite/11605059>

**
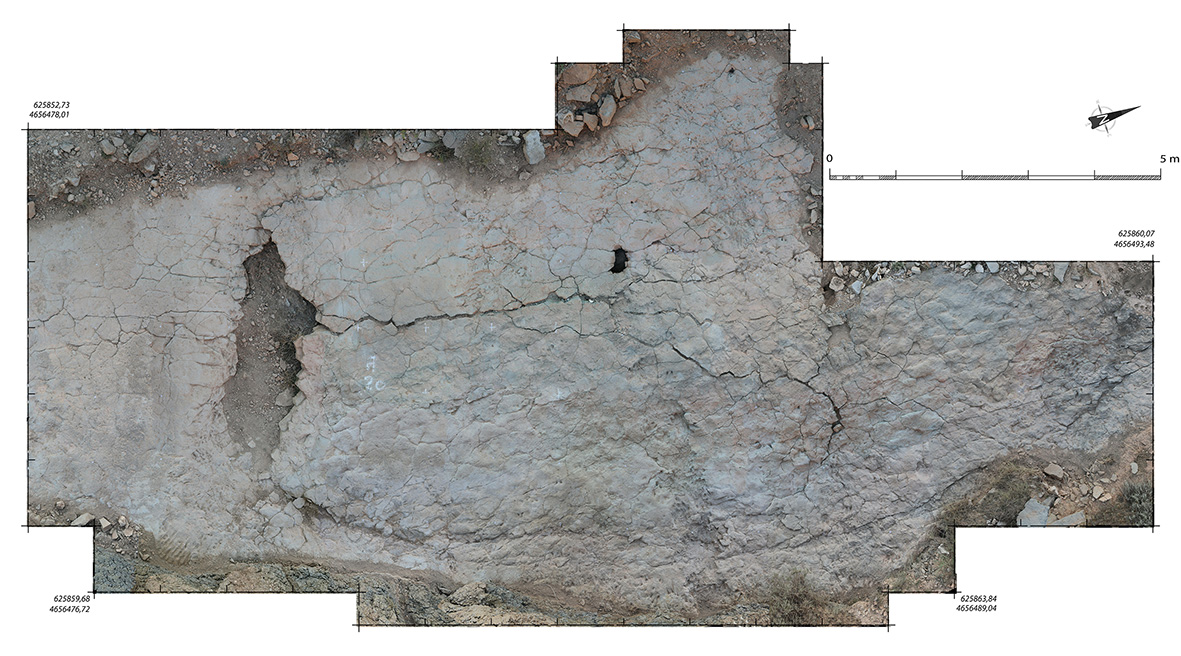
**
